# Insights Into Microbially Induced Salt Tolerance and Endurance Mechanisms (STEM) in Plants

**DOI:** 10.3389/fmicb.2020.01518

**Published:** 2020-08-26

**Authors:** Manoj Kaushal

**Affiliations:** Plant Production and Plant Health, International Institute of Tropical Agriculture (IITA), Dar es Salaam, Tanzania

**Keywords:** salt stress, microbes, ion transporters, signal transduction, aquaporins, photosynthesis

## Abstract

Salt stress threatens the achievement of sustainable global food security goals by inducing secondary stresses, such as osmotic, ionic, and oxidative stress, that are detrimental to plant growth and productivity. Various studies have reported the beneficial roles of microbes in ameliorating salt stress in plants. This review emphasizes salt tolerance and endurance mechanisms (STEM) in microbially inoculated (MI) plants that ensure plant growth and survival. Well-established STEM have been documented in MI plants and include conglomeration of osmolytes, antioxidant barricading, recuperating nutritional status, and ionic homeostasis. This is achieved via involvement of P solubilization, siderophore production, nitrogen fixation, selective ion absorption, volatile organic compound production, exopolysaccharide production, modifications to plant physiological processes (photosynthesis, transpiration, and stomatal conductance), and molecular alterations to alter various biochemical and physiological processes. Salt tolerance and endurance mechanism in MI plants ensures plant growth by improving nutrient uptake and maintaining ionic homeostasis, promoting superior water use efficiency and osmoprotection, enhancing photosynthetic efficiency, preserving cell ultrastructure, and reinforcing antioxidant metabolism. Molecular research in MI plants under salt stress conditions has found variations in the expression profiles of genes such as *HKT1*, *NHX*, and *SOS1* (ion transporters), *PIPs* and *TIPs* (aquaporins), *RBCS, RBCL* (RuBisCo subunits), *Lipoxygenase2* [jasmonic acid (JA) signaling], ABA (abscisic acid)-responsive gene, and *APX, CAT*, and *POD* (involved in antioxidant defense). Proteomic analysis in arbuscular mycorrhizal fungi-inoculated plants revealed upregulated expression of signal transduction proteins, including Ca^2+^ transporter ATPase, calcium-dependent protein kinase, calmodulin, and energy-related proteins (NADH dehydrogenase, iron-sulfur protein NADH dehydrogenase, cytochrome C oxidase, and ATP synthase). Future research should focus on the role of stress hormones, such as JA, salicylic acid, and brassinosteroids, in salt-stressed MI plants and how MI affects the cell wall, secondary metabolism, and signal transduction in host plants.

## Introduction

**FIGURE 1:**
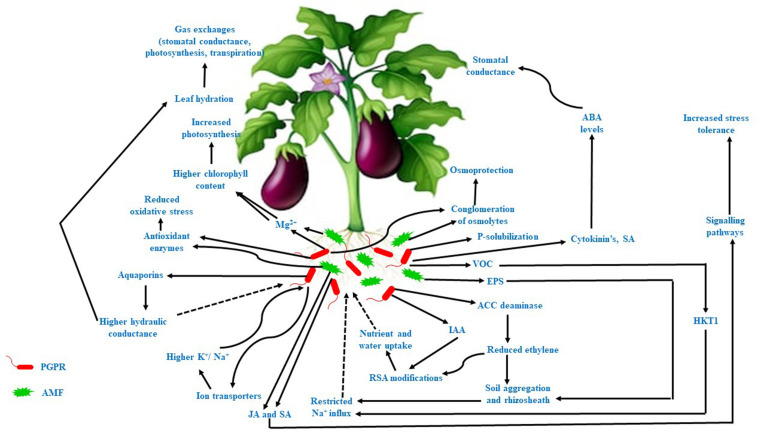
STEM exhibited by PGPR and AMF under salt stress.

Salinity or salt stress is a major threat to agricultural productivity and global food security. It can affect plant growth and development and thus reduce the biomass productivity of plants in arid and semiarid regions. Salt stress is detrimental to plant growth because it induces osmotic and ionic stress in plants, leading to reduced water uptake, transpiration, photosynthesis, and disrupted ionic homeostasis. Moreover, increased levels of reactive oxygen species (ROS) cause oxidative stress, which damages DNA, proteins, and membranes (Liu et al., 2016). Recent studies have confirmed that microbes can induce salt tolerance and endurance mechanisms (STEM) in plants (Table 1) to enable growth and development under harsh stress conditions (Porcel et al., 2016; Barnawal et al., 2017; Chen et al., 2017; Sapre et al., 2018; Yasin et al., 2018; Jia et al., 2019). The various functions of STEM mediating this process can be summarized as follows: (i) conglomeration of osmolytes to abate osmotic stress (Hajiboland et al., 2010; Talaat and Shawky, 2011; Evelin and Kapoor, 2014; Elhindi et al., 2017; Wu et al., 2017; Garg and Bharti, 2018; Hashem et al., 2018); (ii) antioxidant barricading to block oxidative stress (Bharti et al., 2016; Qin et al., 2016; Chang et al., 2018; Chu et al., 2019; Ye et al., 2019); (iii) recuperating nutritional status and ionic homeostasis through P solubilization, siderophore production, nitrogen fixation, ion transporter activity, and exopolysaccharide (EPS) production (Porcel et al., 2016; Elhindi et al., 2017; Zhou et al., 2017; Chang et al., 2018); (iv) physiological modifications in the plant (Barnawal et al., 2017; Chen et al., 2017; Elhindi et al., 2017; Hashem et al., 2018; Ren et al., 2018); and (v) molecular modification of stress-responsive gene expression (Barnawal et al., 2017; Yasin et al., 2018; El-Esawi et al., 2019; Jia et al., 2019). Plant growth-promoting rhizobacteria (PGPR) have been reported to have mitigative effects on the growth of pepper (Yasin et al., 2018), wheat (Bharti et al., 2016; Barnawal et al., 2017), soybean (Khan et al., 2019), oat (Sapre et al., 2018), Panax (Sukweenadhi et al., 2018), and maize (Chen et al., 2016) under salt stress conditions. Similarly, colonization by arbuscular mycorrhizal fungi (AMF) also ameliorated the effects of salt stress in wheat (Fileccia et al., 2017), rice (Porcel et al., 2016), watermelon (Ye et al., 2019), and cucumber (Hashem et al., 2018). The STEM exhibited by PGPR and AMF are illustrated in Figure 1.

**TABLE 1:** STEM in various plant species under salt stress.

**Microbial species**	**Plant**	**STEM in host plants**	**References**
*Enterobacter* spp. EJ01	*Arabidopsis thaliana*	IAA, increased expression of APX, salt stress-responsive genes such as *DREB2b*, *RD29A*, *RD29B*, and *RAB18* in *Arabidopsis*	Kim et al., 2014
*Claroideoglomus etunicatum*	*Oryza sativa*	Increased net photosynthetic rate, stomatal conductance, and transpiration rate	Porcel et al., 2015
*Bacillus* spp. and *Arthrobacter pascens*	*Zea mays* L.	P solubilization, siderophore production, osmolyte accumulation, and higher antioxidant enzyme activity	Ullah and Bano, 2015
*Klebsiella, Pseudomonas, Agrobacterium*, and *Ochrobactrum*	Groundnut	IAA production, N_2_ fixation, phosphate solubilization, ACC deaminase activity, and HCN production	Sharma et al., 2016
*Dietzia natronolimnaea*	*Triticum aestivum*	Altered ABA signaling cascade upregulated *TaABARE* and *TaOPR1*, which upregulated and increased expression of *TaST* (a salt stress-induced gene) and proline content	Bharti et al., 2016
*Variovorax* spp.	*Pisum sativum*	Improved plant water relations, ion homeostasis, and photosynthesis	Wang et al., 2016
*Pantoea dispersa* PSB3	*Cicer arietinum*	Decreased Na^+^ uptake and elevated chlorophyll and K^+^ uptake as well as relative leaf water levels	Panwar et al., 2016
*Bacillus amyloliquefaciens* SQR9	*Zea mays*	Higher chlorophyll and antioxidant production, Na^+^ exclusion from roots; increased expression of *RBCS*, *RBCL* (RuBisCo subunits), ion transporters (*HKT1*, *NHX1*, and *NHX2*), and *H(C)-Ppase* (encoding HC pumping pyrophosphatase)	Chen et al., 2016
*Enterobacter* spp. UPMR18	*Abelmoschus esculentus*	Increase antioxidant enzyme activities and upregulation of antioxidant pathway genes (CAT, APX, and GR)	Habib et al., 2016
*Bacillus, Marinobacterium, Enterobacter, Pantoea, Pseudomonas, Acinetobacter, Rhizobium*, and *Sinorhizobium*	*Triticum aestivum* L.	IAA and siderophore production	Sorty et al., 2016
*Claroideoglomus etunicatum*	*Oryza sativa*	Reduced Na^+^ root-to-shoot distribution, upregulation of *OsNHX3, OsSOS1, OsHKT2;1*, and *OsHKT1*;5 genes	Porcel et al., 2016
*Funneliformis Mosseae*	*Cicer arietinum*	Improved nutrient uptake, reduced chlorophyll pigment damage, and higher *RUBISCO* activity	Garg and Bhandari, 2016b
*Bacillus subtilis* NUU4 and *Mesorhizobium ciceri* IC53	*Cicer arietinum* L.	Increased proline content and P solubilization and improved nutrient acquisition and symbiotic performance of rhizobia	Egamberdieva et al., 2017
*Bacillus amyloliquefaciens*	*Oryza sativa* L.	Reduced ABA and higher SA, upregulated production of glutamic acid and proline	Shahzad et al., 2017
*F. mosseae* and *R. irregularis*	*Cajanus cajan*	Higher GR, APX, and SOD activity	Pandey and Garg, 2017
*Micrococcus yunnanensis, Planococcus rifietoensi*, and *Variovorax paradoxus*	*Beta vulgaris* L.	N_2_ fixation, IAA and siderophore production, P solubilization, and ACCd activity	Zhou et al., 2017
*Microbacterium oleivorans* KNUC7074, *Brevibacterium iodinum* KNUC7183 and *Rhizobium massiliae* KNUC7586	*Capsicum annum* L.	High total soluble sugar, proline contents, Chl contents, and activity of several antioxidant enzymes	Hahm et al., 2017
*Rhizophagus Irregularis* and *Funneliformis mosseae*	*Triticum durum* Desf.	Improved nutrient use efficiency	Fileccia et al., 2017
*Bacillus amyloliquefaciens* FZB42	*Arabidopsis thaliana*	Upregulated expression of genes correlated to photosynthesis, ROS scavenging, auxin, Na^+^ translocation, and JA signaling	Liu et al., 2017
*Rhizophagus irregularis*	*Robinia pseudoacacia* L.	Improved photosynthesis due to higher expression of three chloroplast genes (*RppsbA*, *RppsbD*, and *RprbcL*) in leaves upregulated expression of three genes (*RpSOS1*, *RpHKT1*, and *RpSKOR*) encoding membrane transport proteins involved in K^+^/Na^+^ homeostasis in roots	Chen et al., 2017
*P. fluorescens* SA8 with kinetin (10 mM)	Black gram	Improvement in water use efficiency, gas exchange, and photosynthetic content	Yasin et al., 2018
*Funneliformis mosseae* and *Diversispora versiformis*	*Chrysanthemum morifolium*	Enhanced uptake root N	Wang et al., 2018
*Arthrobacter nitroguajacolicus*	*Triticum aestivum* L.	Higher expression of genes such as Cytochrome P450s, APX, Oligopeptide transporters (OPTs), ATP binding cassette (ABC) transporters, Sugar/inositol transporter, ATPase, and ion transporter	Safdarian et al., 2019
*C. etunicatum, Rhizoglomus intraradices*, and *G. mosseae*	*Cucumis sativus*	Elevated K, Ca, Mg, Fe, Zn, Mn, and Cu content Reduced Na content, higher total phenol as well as activities of SOD, CAT, APX, and GR	Hashem et al., 2018
*Paenibacillus yonginensis* DCY84T	*Panax ginseng*	Higher nutrient availability and expression of salt-defense-related genes viz. ABA synthesis genes, ROS scavenging genes, and ion-pump-related genes	Sukweenadhi et al., 2018
*Klebsiella* spp.	*Avena sativa*	*rbcL* and *WRKY1* altered expression levels	Sapre et al., 2018
*Rhizophagus irregularis*	*Elaeagnus angustifolia* L.	Higher activities of SOD, CAT, and APX, increased uptake of K^+^, Ca^2+^, and Mg^2+^	Chang et al., 2018
*Glomus tortuosum*	*Zea mays*	Increased Chl content, RuBisCO activity, and net photosynthetic rate	Xu et al., 2018
*Funneliformis mosseae*	*Citrullus lanatus* L.	Reduced expression level of PPH (chlorophyll degradation), higher net photosynthesis rate and increased expression of antioxidant response-related genes Cu-Zn SOD, CAT, APX, and GR	Ye et al., 2019
*Azospirillum lipoferum* FK1	*Cicer arietinum* L.	Improved nutrient acquisition, photosynthetic pigment synthesis, and osmolyte content, and higher antioxidant defense	El-Esawi et al., 2019
*Pseudomonas* spp.	*Arabidopsis thaliana*	Upregulation in *LOX2*	Chu et al., 2019
*Rhizophagus intraradices* and *Funneliformis mosseae*	*Arundo donax* L.	Improved nutrient use efficiency	Romero-Munar et al., 2019
*Rhizophagus irregularis*	*E. angustifolia*	Improved efficiency of photosystem II and enhanced expression of proteins involved in secondary metabolism, antioxidant defense, and signal transduction	Jia et al., 2019

## Conglomeration of Osmolytes and Water Homeostasis to Abate Osmotic Stress

In its initial phase, salt stress can be referred to as physiological drought because elevated ion levels during salt stress change the soil texture to reduce soil porosity and decrease water uptake. Osmolyte conglomeration is a major STEM that improves water uptake in microbially inoculated (MI) plants. This reduces the water potential by accumulating osmolytes, such as amino acids (proline), amines (e.g., glycinebetaine, polyamines), sugars, and organic acids (e.g., oxalate, malate). In addition to osmotic adjustment, these osmolytes are responsible for conserving membrane integrity, protein stability, and ROS scavenging to ultimately promote their positive effects on plant physiological functions, such as growth, photosynthesis, and crop yield, during salt stress (Zou et al., 2013). Proline and glycinebetaine enhance protein and membrane stabilization to impart osmoprotection to salt-stressed plants. However, contrasting results regarding proline production have been reported in MI and non-inoculated (NI) plants. Increased proline content in NI plants compared to AMF-inoculated (AI) plants can indicate higher stress conditions. AI plants show decreased proline content because microbial colonization helps the plant mitigate the stress. Some studies have suggested that proline accumulation is a salinity stress indicator rather than a consequence of mycorrhizal colonization (Sheng et al., 2011; Echeverria et al., 2013; Evelin et al., 2013; Evelin and Kapoor, 2014); however, other studies have found higher proline accumulations caused by AM colonization (Hajiboland et al., 2010; Talaat and Shawky, 2011; Garg and Baher, 2013; Elhindi et al., 2017; Hashem et al., 2018). Higher proline levels have been observed in PGPR-inoculated maize (Ullah and Bano, 2015), *Gladiolus* (Damodaran et al., 2014), *Mentha* (Bharti et al., 2014), *Chrysanthemum* (Wang et al., 2018), and *Panax* (Sukweenadhi et al., 2018). Increased proline levels in MI plants can be due to (i) upregulated expression P5CS, a gene involved in proline synthesis; (ii) increased efficiency of the enzymes P5CS and glutamate dehydrogenase (involved in glutamate synthesis) given that proline is synthesized from glutamate; and (iii) arrest of proline dehydrogenase (responsible for proline degradation) (Abo-Doma et al., 2016). In the nodules of AI-inoculated pigeon pea plants, reduced activity of trehalase (trehalose degrading enzyme) and increased activity of trehalose-6-P synthase and trehalose-6-phosphatase (enzymes involved in the biosynthesis of trehalose) led to higher trehalose levels (Garg and Pandey, 2016). Higher concentrations of acetic, malic, citric, oxalic, and fumaric acids were observed in AI maize plants compared to NI plants and led to enhanced salinity tolerance (Sheng et al., 2011). Arbuscular mycorrhizal fungi treatment alters polyamine levels in plants to impart stress tolerance (Evelin et al., 2013; Talaat and Shawky, 2013). Evelin et al. (2013) observed increased spermidine and spermine (Spd + Spm)/putrescine (Put) ratios in AI fenugreek plants compared to NI plants. Salinity tolerance in MI plants is advantageous and can correlate with their ability to join DNA, proteins, and phospholipids. Polyamine levels were modulated in response to mycorrhizal colonization in two cultivars of wheat (Sids 1 and Giza 168) under saline conditions. AM colonization led to higher putrescine but lower spermidine and spermine levels in Giza 168; however, in Sid 1, a reduction in putrescine and an increase in spermidine and spermine levels were reported (Talaat and Shawky, 2013). The accumulation of total soluble sugars (TSS), such as glucose, sucrose, and maltose, during salt stress in MI plants is another mode of STEM via osmotic adjustment. Conversion of starch into dextrins and maltose is accompanied by a- and b-amylases, respectively. Researchers have confirmed that enhanced salt stress tolerance in MI plants is due to higher TSS accumulation (Talaat and Shawky, 2011; Liu et al., 2016; Garg and Bharti, 2018; Zhu et al., 2018). Arbuscular mycorrhizal fungi inoculation modifies leaf sucrose and proline metabolism by regulating the enzymatic activities responsible for sucrose and proline metabolism to enhance osmotic tolerance in the host plant (Wu et al., 2017). Elevated TSS content can be caused by increased photosynthesis, amylase activity, and increased organic acid levels (Yu et al., 2015; Garg and Bharti, 2018; Zhu et al., 2018). Studies in MI chickpea plants have demonstrated that increased salt tolerance can be achieved by the synthesis of proline, glycinebetaine, and increasing TSS (Qurashi and Sabri, 2012; Upadhyay and Singh, 2015). Elevated glycinebetaine levels enhance salinity tolerance in rhizobacterially primed rice plants (Jha et al., 2011), AI wheat (Talaat and Shawky, 2011), and maize (Sheng et al., 2011). According to Rangel (2011), bacteria grown under glucose concentration have low cAMP levels, but when grown under carbon starvation, bacteria show higher cAMP levels. However, the converse is true for eukaryotes. This aspect needs to be addressed in relation to plant microbial crosstalk under salt stress conditions. Future research should focus on unraveling the molecular mechanisms underlying the role of microbes in promoting osmotic adjustment during salt stress.

## Antioxidant Barricading to Caulk the Oxidative Stress

The hyperosmotic and hyperionic conditions present during salt stress disrupt cellular redox homeostasis by disrupting the equilibrium between the generation and elimination of ROS, leading to oxidative stress as a secondary stress. Reactive oxygen species target various biomolecules, including nucleic acids, proteins and fatty acids, to alter cellular function, cause DNA damage, reduce membrane fluidity, cause lipid peroxidation, and affect enzymatic activity. It is evident that ROS create oxidative stress; however, they are also involved in ethylene accumulation, auxin biosynthesis, and many signaling events (Kaushal, 2019). Thus, it is essential that an equilibrium is maintained between ROS generation and ROS scavenging systems to balance oxidative damage while managing endogenous signaling events. Plants are equipped with a robust antioxidant system consisting of enzymatic [superoxide dismutase (*SOD*), catalase (*CAT*), ascorbate peroxidase (*APX*), monodehydroascorbate reductase (*MDHAR*), and glutathione reductase (*GR*)] and non-enzymatic (cysteine, carotenoids, glutathione, tocopherols, and ascorbate) constituents. The induction of the antioxidative defense system has been shown to be another STEM activated in MI plants to abate salt stress (Talaat and Shawky, 2011; Li et al., 2012; Bharti et al., 2016; Chang et al., 2018). Higher antioxidant activities have been observed in AI tomato (Hajiboland et al., 2010; Latef and Chaoxing, 2011), *Sesbania* (Abd Allah et al., 2015), pigeon pea (Pandey and Garg, 2017), and *Cucumis* (Hashem et al., 2018) plants during salt stress. Wu et al. (2016), while investigating the impact of AMF colonization and salt stress on male and female *Populus cathayana* seedlings, observed significant increases in the activities of *SOD* and *CAT* in the roots of AI-colonized plants compared to those of NI plants; however, *CAT* activity was similar in the leaves of AI and NI plants. Three-way ANOVA revealed that the activities of *SOD*, *POD*, and *CAT* in roots were influenced by AMF × salt × sex, salt × sex, and AMF × sex, AMF × sex, and AMF × sex × salt, respectively. This demonstrated that the activities of different antioxidant enzymes were variably affected by the interactions between salt stress, gender, and AMF. A significant increase in the expression levels of genes related to the antioxidant response, such as Cu-Zn *SOD*, *CAT*, *APX*, and *GR*, was reported during salinity alkalinity stress and was further enhanced by AMF inoculation, thereby enabling host watermelon plants to cope with the stress (Ye et al., 2019). Higher root, stem, and leaf biomass was observed in AI seedlings of *Elaeagnus angustifolia* L. during salt stress, which was attributed to the increased activities of *SOD*, *CAT*, and *APX* in the leaves relative to those of NI plants (Chang et al., 2018). In addition to increased activity and expression levels of *CAT*, *GPOX*, *APX*, *SOD*, *MDHAR*, *DHAR*, and *GR*, AI *Cicer arietinum* plants also demonstrated enhanced levels of GSG, GSSH, and total glutathione (Garg and Bhandari, 2016a). Similar observations of STEM involving higher antioxidant barricading have been reported in rhizobacterially inoculated (RI) plants (Gururani et al., 2013; Kim et al., 2014; Tewari and Arora, 2014; Ullah and Bano, 2015; Bharti et al., 2016; Kaushal and Wani, 2016a; Singh and Jha, 2017). A significant increase in the specific activities of *APX* (1.4 times), *SOD* (2.4 times), and *CAT* (1.8 times) was observed in RI *Solanum* plants under salt stress, and antioxidant enzyme activity was positively correlated with the mRNA expression levels of the corresponding genes encoding these enzymes (Gururani et al., 2013). In a similar study, higher activities of *APX* and *CAT* and upregulation of antioxidant pathway genes (*CAT*, *APX*, and *GR*) were observed in *Enterobacter* spp. UPMR18-colonized okra plants, improving the physiological performance and salt tolerance of the plants (Habib et al., 2016). In *Arabidopsis thaliana* roots colonized with *Burkholderia phytofirmans* PsJNA, genes involved in ROS quenching (*APX2*) were significantly more transcribed, helping the plant to abate oxidative stress (Pinedo et al., 2015). In PGPR *Dietzia natronolimnaea* STR1-colonized wheat plants, the gene expression of certain antioxidant enzymes (*APX*, *MnSOD*, *CAT*, *POD*, *GPX*, and *GR*) was enhanced to alleviate salt stress (Bharti et al., 2016). Higher activities of antioxidant enzymes in MI plants can correlate with improved nutritional status (Cu, Mn, and Fe) because these enzymes are in fact metalloenzymes, and their activities are therefore governed by the presence of these nutrients (Kohler et al., 2009). Moreover, the activity of these enzymes also depends on the plant species, microbes, and stress timing. AI plants were able to abate the effect of salt stress by enhancing the activity of antioxidant enzymes, including *SOD*, *CAT*, and *APX*, and increasing ascorbic acid levels, which correlates with lower lipid peroxidation and electrolyte leakage. Zn, Cu, Mn, and Fe serve as co-factors for *SOD* isozymes, and their increased uptake in AI plants was able to boost *SOD* activity (Hashem et al., 2018).

## Salinity Tolerance Through Recuperation of Nutritional Status and Ionic Homeostasis

Salinity can lead to altered nutritional status and ionic homeostasis in plants, hampering the plant’s productivity. Microbial colonization during stress improves the physiological performance of plants with an additional STEM involving enhanced nutrient uptake and selective ion absorption and translocation (Evelin et al., 2012; Bharti et al., 2014; Kang et al., 2014b; Tewari and Arora, 2014; Porcel et al., 2016; Hashem et al., 2018). The availability of nutrients such as P, N, Mn, and Fe is restricted in saline soils. Microbial inoculation simplifies the process of acquiring these nutrients for plants under salt stress conditions to promote plant health and productivity. Plant growth-promoting rhizobacteria strains act as phosphate-solubilizing rhizobacteria to increase the uptake and availability of P to plants (Prasad et al., 2015; Etesami, 2018). Salt tolerance was enhanced in PGPR-colonized *Mentha* (Bharti et al., 2014), wheat (Upadhyay and Singh, 2015), *Chrysanthemum* (Zhou et al., 2017), and groundnut (Shukla et al., 2012) plants due to enhanced phosphate nutrition. Improved phosphorus absorption was found in AI plants under mycorrhizal inoculation, even under salt stress conditions (Sharifi et al., 2007; Al-Khaliel, 2010; Bowles et al., 2016). It is postulated that phosphate is absorbed and converted to polyphosphate by the extraradical mycelium. Recent studies have demonstrated the involvement of AM aquaporins in the translocation of polyphosphate via mycorrhizal hyphae. Thus, adequate P uptake in AI plants helps selective ion absorption, limits toxic ions in vacuoles, and preserves membrane integrity (Evelin et al., 2012) to reverse the effects of salt stress. However, it has been suggested that enhanced growth in MI plants was caused by improved photosynthesis and WUE (water use efficiency) rather than by the increased mineral uptake (Ruiz-Lozano et al., 1996; Garg and Bhandari, 2016b; Chen et al., 2017). Moreover, Feng et al. (2002) reported that salt tolerance in AMF plants was conferred by increased soluble sugar accumulation rather than P levels. Rhizobacterial strains often secrete siderophores to cope with iron deficiency in plants surrounded by saline soil. Siderophores are high affinity low molecular weight Fe (III) chelators that scavenge Fe^3+^ to form an iron–siderophore complex that can be readily solubilized to increase iron availability to plants. Recent studies have reported enhanced salinity tolerance in rhizobacterially colonized plants resulting from siderophore production (Shukla et al., 2012; Sorty et al., 2016; Navarro-Torre et al., 2017; Zhou et al., 2017). Rhizobacterial strains that produce EPSs improve plant growth under salt stress through rhizosheath development around the plant roots, which limits the Na^+^ influx inside the stele (Ashraf et al., 2004). In wheat plants, EPS production by PGPR ameliorated salt stress via fusion of Na^+^ ions to EPS, leading to enhanced plant nutrition and growth. EPS adheres to soil particles to build macro aggregates that stabilize soil structure and ultimately improve its hydraulic water holding and cation exchange capacity (Upadhyay et al., 2011a, b).

Salt stress impedes plant growth through elevated levels of Na^+^ and a lower K^+^/Na^+^ ratio. *Bacillus*-colonized *Gladiolus* plants displayed increased K^+^ uptake relative to Na^+^, reducing the Na^+^/K^+^ ratio under saline conditions (Damodaran et al., 2014). Rhizobacterially inoculated maize plants improved ionic balance by enhancing root K^+^ uptake and Na^+^ exclusion to confer salt tolerance (Rojas-Tapias et al., 2012). In an interesting study by Pinedo et al. (2015), PGPR *B. phytofirmans* PsJN-primed *Arabidopsis* plants were found to sustain salt stress conditions, and this correlated with altered expression of genes involved in ionic equilibrium, such as *Arabidopsis* K^+^ Transporter1 (*KT1*), High-Affinity K^+^ Transporter1 (*HKT1*), Sodium Hydrogen Exchanger2 (*NHX2*), and *Arabidopsis* Salt Overly Sensitive 1 (*SOS1*). *Bacillus subtilis* GB03-colonized *Puccinellia tenuiflora* displayed increased expression of *PtHKT1* and *PtSOS1* and downregulated expression of *PtHKT2* genes in plant roots. Thus, limiting Na^+^ ion uptake in roots and their subsequent translocation reduced Na^+^ ion accumulation (Niu et al., 2016). Higher Na^+^ concentrations in rhizospheres provide strong competition against K^+^ ions, elevating the Na^+^/K^+^ ratio and causing higher stress by disrupting metabolic and physiological processes. Higher K^+^/Na^+^ ratios in AI plants is caused by the controlled translocation of Na^+^ ions to aboveground tissues of host plants and their accumulation in vacuoles. Salinity caused an escalation in the Na^+^ shoot to root ratio levels; however, this ratio was reduced in AMF seedlings (Evelin et al., 2012; Porcel et al., 2016). Additionally, it has been confirmed that enhanced K^+^ absorption and reduced Na^+^ transportation to shoot tissues lead to higher K^+^/Na^+^ ratios in AI plants during salt stress (Sharifi et al., 2007; Talaat and Shawky, 2011; Estrada et al., 2013a), which serve to preserve enzymatic processes and protein synthesis.

AMF acts as a primary barrier for absorption of ions during fungal nutrient uptake from soil or their transportation to host plants. This is attributed to the capability of AMF to retain these minerals in intraradical mycelium and vesicles via ionic accumulation in vacuoles (Mardukhi et al., 2011). This type of selective ion absorption (higher K^+^, Mg^2+^, and Ca^2+^ uptake; reduced Na^+^ uptake) leads to higher K^+^/Na^+^, Ca^2+^/Na^+^, and Mg^2+^/Na^+^ ratios in *Rhizophagus intraradices*. In addition, AI plants have increased Na^+^ content, up to a certain limit, which is then reduced at higher salinity levels, suggesting an AMF-induced buffering effect on Na^+^ uptake (Hammer and Rillig, 2011). AI-colonized wheat plants showed a significant increase in yield at various salinity levels that correlated to higher levels of N, P, and K, and reduced levels of Na^+^ in the leaves (Talaat and Shawky, 2013). Arbuscular mycorrhizal fungi improved K^+^ ion retention in maize plant tissues following upregulated expression of *ZmAKT2* and *ZmSKOR* (Estrada et al., 2013b). A significant increase in K^+^ levels and decrease in Na^+^ levels were observed in AMF plants, suggesting selective uptake of K^+^ but not Na^+^ into the xylem of plant roots, which thus increases the K^+^/Na^+^ ratio under salt stress conditions to improve plant growth (Elhindi et al., 2017). Nitrogen assimilation in AI host plants is more efficient due to nitrate assimilation and higher enzyme production in the extraradical mycelia (Evelin et al., 2009; Kapoor et al., 2013). Enzymatic activities and protein synthesis were preserved in AI plants by increased nitrate reductase activity (caused by the elevated nitrate assimilation), increased K^+^ accumulation, and an improved K^+^/Na^+^ ratio (Talaat and Shawky, 2014). Wang et al. (2018) reported enhanced N uptake by plant roots in AI plants, which increased root length and root and shoot weight, to be the major mechanism underlying enhanced salt tolerance in *Diversispora versiformis-*colonized *Chrysanthemum morifolium*. Increased absorption of nutrients including Fe, K, Ca, Fe, Zn, and Mg, but restricted Na and Cl uptake in AI-colonized plants has been reported as a STEM to maintain ionic equilibrium and mitigate the effects of salt stress (Evelin et al., 2012; Kapoor et al., 2013). During salt stress, increased rhizospheric Na^+^ levels obstruct Ca^2+^ absorption and therefore disrupt the Ca^2+^:Na^+^ ratio of host plants, ultimately decreasing their hydraulic conductivity and disturbing Ca^2+^ signaling. Improving nutritional status is essential for conserving membrane integrity in AI plants. Mycorrhizal association increased Ca^2+^ and Mg^2+^ absorption by plant roots, even under soil salinity (Giri and Mukerji, 2004; Sharifi et al., 2007). Ca^2+^ levels were increased in *Piriformospora indica -*colonized barley plants, leading to the activation of signal transduction pathways to enhance stress tolerance in host plants (Alikhani et al., 2013). Given that Mg^2+^ is centrally located in the chlorophyll molecule, deceases in its uptake can reduce chlorophyll content and photosynthesis and eventually hamper plant growth. In AI host plants, an increase in Mg^2+^ uptake increases chlorophyll concentration to boost photosynthesis and plant performance while under stress conditions (Abdel Latef and Chaoxing, 2014). Enhanced salt tolerance in *Rhizophagus irregularis-*colonized *E. angustifolia* seedlings was correlated with increased K^+^, Ca^2+^, and Mg^2+^ uptake. Additionally, AM symbioses altered root architecture, and extraradical mycelia improved mineral uptake. K^+^ accumulation was increased in the roots and leaves of AI seedlings, leading to an enhanced K^+^/Na^+^ ratio in the plants and suggesting a STEM in plants (Chang et al., 2018).

## Modifications in Plant Physiological Status

Microbially induced STEM includes phytohormonal modifications and alterations in other physiological processes, such as gas exchange, photosynthesis, and nutrient and water uptake. Various studies have established the role of microbes in alleviating the negative effects induced by salt stress on plant physiological performance (Porcel et al., 2015; Chen et al., 2017).

### Phytohormonal Modulations

Microbes can promote plant growth during salt stress by altering the hormonal status of NI plants. Various phytohormones, including auxins, gibberellins, cytokinin, ethylene, ABA, JA, and SA, are involved in signaling events during plant–microbe interactions that can rescue plants under stress conditions. Numerous studies have reported roles for bacteria in phytohormonal modulations in response to salt stress, but there are relatively few studies related to AMF-mediated phytohormonal salt stress tolerance in host plants. Production of auxins, such as indole acetic acid (IAA), by PGPR strains has been well documented in RI plants and ensures plant survival during salt stress (Egamberdieva, 2009; Jha et al., 2012; Sharma et al., 2016; Sorty et al., 2016; Navarro-Torre et al., 2017). Rhizobacterially inoculated wheat plants showed elevated IAA levels in their rhizospheres compared to NI plants, which led to improved plant growth and survival under stress conditions (Tiwari et al., 2011). Higher root growth was reported in *Pseudomonas chlororaphis* TSAU13-primed wheat seedlings, tomato, and cucumber plants. IAA production by PGPR *P. chlororaphis* TSAU13 altered phytohormonal levels in plants and consequently enhanced stress tolerance compared to NI plants (Egamberdieva and Kucharova, 2009; Egamberdieva, 2012). Higher IAA levels in wheat plants inoculated with PGPR strains *Arthrobacter protophormiae* and *D. natronolimnaea* enabled host plants to survive salt stress (Barnawal et al., 2017). Chickpea plants co-inoculated with IAA-synthesizing rhizobacterial strains (*B. subtilis* NUU4 and *Mesorhizobium ciceri* IC53) exhibited increased root and shoot biomass, along with enhanced nodule formation relative to untreated plants and plants treated solely with *M. ciceri* IC53 (Egamberdieva et al., 2017). Similar observations of increased IAA levels have been reported in PGPR inoculated peanut (Sharma et al., 2016), barley (Cardinale et al., 2015), and wheat (Singh and Jha, 2016; Sorty et al., 2016). Plant growth-promoting rhizobacteria can modulate GA levels in RI plants (Kang et al., 2014a, b). Increased endogenous levels of gibberellins in *Pseudomonas putida* H-2-3 primed soybean plants (Kang et al., 2014b) and improved plant growth in salt stress conditions. Although cytokinin production is common in microbial strains and imparts stress tolerance to inoculated plants (Liu et al., 2013), very few studies have reported the role of cytokinin in MI plants. Higher proline content, in addition to increased shoot and root biomass, has been reported during salt stress in soybean plants inoculated with the cytokinin synthesizing rhizobacterial strains *Arthrobacter*, *Azospirillum*, and *Bacillus* (Naz et al., 2009). Accelerated ethylene synthesis above threshold values while under stress conditions restricts plant growth by negatively affecting root development and seed germination. However, rhizobacterial strains possessing *ACCd* can limit ethylene levels by cleaving the ethylene precursor, 1-aminocyclopropane-1-carboxylate (ACC), to produce ammonia and a-ketobutyrate. Various studies have reported plant growth promoted by *ACCd-*producing PGPR strains that also alleviates salt stress (Ali et al., 2014; Bharti et al., 2014). Nadeem et al. (2009) reported that *ACCd*-producing PGPR strains (*Pseudomonas fluorescens*, and *Enterobacter* spp.) improved the mineral status of maize plants, thereby helping them to counteract salt stress. Priming chickpea plants with *ACCd*-producing *Pantoea dispersa* PSB3 led to decreased Na^+^ uptake and elevated chlorophyll levels and relative leaf water levels, resulting in increased pod number and weight, biomass, and seed weight during salt stress (Panwar et al., 2016). Pea plants primed with *ACCd*-producing *Variovorax paradoxus* 5C-2 displayed root to shoot K^+^ ionic flow and Na^+^ ion root deposition, which led to elevated K^+^/Na^+^ ratios in the shoots. Moreover, a higher photosynthesis rate and decreased stomatal resistance caused the plant biomass to increase, thus enhancing stress tolerance (Wang et al., 2016). *Enterobacter* spp. UPMR18-treated okra plants displayed higher antioxidant enzyme activities and increased transcription of ROS pathway genes (Habib et al., 2016). Abscisic acid is a major stress phytohormone capable of alleviating abiotic stress by mediating the important physiological processes of stomatal opening and photosynthesis. Enhanced root and shoot growth were observed in rice plants treated with ABA-producing endophytic bacteria (Shahzad et al., 2017). *D. natronolimnaea* STR1-treated wheat plants showed enhanced salt tolerance via alteration of the ABA signaling cascade, which was confirmed by upregulation of *TaABARE* (ABA-responsive gene) and *TaOPR1* (12-oxophytodienoate reductase 1) genes (Bharti et al., 2016). Treatment of maize plants with *Bacillus amyloliquefaciens* SQR9 conferred salt stress tolerance; treated plants counteracted increased ABA levels and exhibited enhanced chlorophyll levels, glutathione content, and K^+^/Na^+^ ratios (Chen et al., 2016). A significant increase in JA content and decrease in SA were recorded during salt stress in soybean plants (Kang et al., 2014b). Increased nutrient acquisition and salt stress tolerance were observed in maize plants upon inoculation with SA-synthesizing *Serratia marcescens* (Lavania and Nautiyal, 2013). Rice plants treated with *B. amyloliquefaciens* RWL-1 have been shown to have elevated endogenous SA levels and lower endogenous JA and ABA levels compared to plants treated with GA3 and water (Shahzad et al., 2016). Treatment with *B. megaterium* highlighted the role of *JA-Ile* turnover in the recovery of *Arabidopsis* plants from salt stress (Erice et al., 2017). Elevation of photosynthetic pigments and shoot biomass was reported in soybean plants under salt stress conditions in response to gibberellins produced by *Aspergillus fumigatus* (Khan et al., 2011). Decreased ABA production was found to regulate transpiration rate in cucumber plants colonized by AMF. However, JA and SA synthesis was increased upon AMF inoculation, which decreased oxidative damage to enhance salt stress tolerance (Hashem et al., 2018). In another study, higher levels of JA and its precursor, OPDA, were found in *Digitaria eriantha* colonized by *R. irregularis* under salt stress conditions, demonstrating the key role of JA in conferring salt tolerance to plants (Pedranzani et al., 2016). Strigolactones are the latest class of phytohormones found to be involved in adventitious root formation, reproductive development, and stress responses. A positive correlation was found between ABA and strigolactones in AMF-colonized *Sesbania*. Raised ABA levels caused higher H_2_O_2_ production, which led to increased SA synthesis and subsequently protected mycorrhizal plants from salt stress (Ren et al., 2018). Aroca et al. (2013) reported that strigolactone production was induced in AM-colonized lettuce plants under salt stress conditions and was correlated to ABA. More research studies targeting the role of AMF in altering phytohormonal levels in plants during salt stress are needed.

### Improved Photosynthesis and Other Physiological Changes

Arbuscular mycorrhizal fungi-inoculated plants sustain higher chlorophyll and carotenoid levels through enhanced Mg^2+^ ion uptake (Evelin et al., 2012; Hashem et al., 2015), which is otherwise restricted by salt stress. Physiological changes of AI and RI plants also included a higher quantum yield of PSII and increased net photosynthetic rate (Talaat and Shawky, 2014; Chen et al., 2016, 2017; Hidri et al., 2016; Wang et al., 2016; Yasin et al., 2018) relative to NI or control plants. Glycinebetaine preserves the activities of RuBisCO and rubisco activase involved in CO_2_ fixation and secures PSII pigment-protein complexes (Talaat and Shawky, 2014) to confer salt stress tolerance to plants (Porcel et al., 2016; Hu et al., 2017). Higher RuBisCO activity has been reported in AMF plants (Garg and Bhandari, 2016b; Chen et al., 2017) and is correlated with increased *RprbcL* gene expression (Chen et al., 2017). AM symbiosis alleviates the physiological drought effects on photosynthesis by improving the water status of colonized plants through increases in their leaf area and stomatal conductance (Chen et al., 2017). Arbuscular mycorrhizal fungi can protect the photosystems by reducing non-photochemical quenching in inoculated plants relative to uninoculated ones and thus enhance their photosynthetic efficiency under salt stress conditions (Hu et al., 2017). Higher stomatal conductance and strong photosystem efficiency in AMF-colonized plants reduced photorespiration and lowered ROS production, which subsequently conferred salt stress tolerance (Ruiz-Sánchez et al., 2010). In AM rice plants under salt stress conditions, the enhanced quantum yield of photosystem II and reduced non-photochemical quenching-maintained photosynthesis, transpiration, and stomatal conductance led to increased biomass (Porcel et al., 2015). Ye et al. (2019) reported that AMF alleviated the decrease in the maximum photochemical efficiency of Photosystem II (Fv/Fm), photochemical quenching and increase in non-photochemical quenching (*NPQ*) observed in salt-stressed watermelon plants. Numerous studies have reported higher WUE, stomatal conductance, transpiration rate, and photosynthesis in AI plants exposed to salt stress compared to non-colonized plants (Sheng et al., 2008; Evelin et al., 2012; Elhindi et al., 2017). Wu et al. (2015) investigated the role of AMF in different sexes of *P. cathayana* during salt stress. Fv/Fm was elevated in males compared to females. Moreover, the improved efficiency of photosystem II and the antioxidant machinery in mycorrhizal seedlings alleviated the effects of salt stress. Increases in Fv/Fm, *NPQ*, and *ETR* were observed in the leaves of AI *E. angustifolia* compared to non-mycorrhizal plants under salt stress (Jia et al., 2019). Increased electrical conductivity was noticed in AMF-colonized plants and is caused by the reduced electrolyte permeability of root plasma membranes relative to non-mycorrhizal plant roots (Garg and Manchanda, 2008). Increased hydraulic conductance (Aroca et al., 2007), root system modifications (Campanelli et al., 2013; Alqarawi et al., 2014), and an improved water status were observed in AI plants under salt stress (Chen et al., 2017). Improved water status in AMF plants correlates with the expression of aquaporin genes (*RpPIP1*;*1*, *RpPIP1*;*3*, *RpPIP2*;*1*, *RpTIP1*;*1*, *RpTIP1*;*3*, *RpTIP2*;*1*) in leaves and roots of plants under salt stress; however, expression levels vary according to plant species, salinity levels, and location or tissue of expression (Chen et al., 2017). Higher WUE was observed in sweet basil plants colonized with *Glomus deserticola* compared to control plants under salt stress (Elhindi et al., 2017).

Significant increases in fresh and dry weight, plant height, and chlorophyll content were observed in PGPR-inoculated pepper plants relative to NI plants (Hahm et al., 2017). An increase in chlorophyll levels in PGPR-inoculated plants (Shukla et al., 2012; Bharti et al., 2014) and higher WUE of inoculated capsicum plants enabled them to survive under salt stress conditions (Yasin et al., 2018). Higher chlorophyll production, Na^+^ exclusion from roots, and antioxidant production enhanced salt stress tolerance in maize plants inoculated with *B. amyloliquefaciens* (Chen et al., 2016). Increased photosynthetic rates and decreased stomatal resistance enhanced the plant biomass of PGPR-inoculated pea plants (Wang et al., 2016). An interesting study by Chatterjee et al. (2018) found that treatment of rice plants with ACC deaminase-containing *Brevibacterium linens* RS16B decreased volatile organic compound (VOC) emissions and enhanced photosynthesis by reducing the availability of ACC and ACC oxidase activity, suggesting that research on volatile emissions during salt stress can reveal new insights related to stress severity and the initiation of secondary metabolism with stress progression. Ansari et al. (2019) demonstrated that PGPR inoculation increased root length, chlorophyll pigments, leaf number, relative water content (RWC), stomatal conductance (gs), and photosynthesis rate (Pn) in alfalfa plants under salt stress. *Pseudomonas* inoculation improved leaf number, chlorophyll pigments, nodule number, Na^+^ levels, and K^+^/Na^+^ ratios; however, *Hartmannibacter* inoculation improved carotenoid content, RWC, and K^+^ levels. Higher gs and chlorophyll pigments enhanced Pn in RI alfalfa plants. Inoculation with *Bacillus megaterium* strain A12 ameliorated salt stress in tomato plants by restoring redox homeostasis and photosynthesis to improve plant growth. Higher expression levels of the *PBGD* gene (encodes the enzyme needed for chlorophyll biosynthesis) enhanced chlorophyll content in tomato plants. In addition, reduction of ROS levels upregulated expression of the *PsbA* gene (encodes D1 protein that repairs stress damaged photosystem). Increased cytokinin production diminished the degradation of photosynthetic proteins and elevated the expression levels of genes related to photosystems under stress conditions (Akram et al., 2019).

## Molecular Alterations

Multi-omics approaches, such as metagenomics, metatranscriptomics, and metaproteomics, can be utilized to enhance our understanding of plant behavior under stress conditions. However, it remains a challenge to integrate data from various “omics” tools, although this would be a step toward understanding the complex crosstalk between plants and microbes (Meena et al., 2017). STEM affecting various physiological and biochemical processes under salt stress involve variations in expression levels of genes, including ion transporters, aquaporins, and Δ1-pyrroline-5-carboxylate synthetase (P5CS), and variable levels of antioxidant defense enzymes, late embryogenesis abundant protein photosynthesis, antioxidant defense ionic homeostasis, and other signaling events. However, molecular alterations are categorized as a separate STEM to provide a better understanding, at transcriptional and proteomic levels, of how the plant response to salt stress is influenced.

### Transcriptional Studies

Upregulation of *SOS1*, *NADP-Me2* (NADP-malic enzyme), *EREBP* (ethylene-responsive element binding proteins), and SERK1 (somatic embryogenesis receptor-like kinase) and downregulation of GIG (glucose-insensitive growth) and SNF1 (serinethreonine protein kinase *SAPK4*) expression were observed in *B. amyloliquefaciens* SN13-inoculated rice plants exposed to salt stress. Modulated gene expression mitigated osmotic and ionic stress in plants to improve plant performance under stress conditions (Nautiyal et al., 2013). A significant increase in the transcriptional levels of *AtRSA1* and *AtWRKY8* and decrease in the expression of *AtVQ9* (*AtWRKY8* antagonist) in PGPR-treated *Arabidopsis* plants suggested enhanced plant performance under stress conditions resulting from microbial colonization. *AtRSA1* forms a complex with the *AtRITF1* transcription factor to regulate Na^+^ ion homeostasis and ROS detoxification during salt stress; *AtWRKY8* and *AtVQ9* are also involved in preserving ion homeostasis at reduced Na^+^/K^+^ ratios in the cytosol (Sukweenadhi et al., 2015). Upregulated expression of genes involved in stress responses [e.g., *RAB18* (*LEA*), *RD29B* regulons of ABA-responsive elements], proline biosynthesis (e.g., *P5CS1* and *P5CS2*), and *MPK3* and *MPK6* stress responses have also been reported (Kim et al., 2014). Upregulated expression of the *TaCTR1* (Serine/Threonine protein kinase–ethylene responsive) and *TaDREB2* (encodes a transcription factor enhancing abiotic stress tolerance in plants) genes was reported in wheat plants inoculated with PGPR strains (Kaushal and Wani, 2016b; Barnawal et al., 2017). *B. subtilis* GB03-inoculated *P. tenuiflora* plants showed less Na^+^ accumulation in response to upregulated expression of *PtHKT1*;*5* (involved in the acquisition of Na^+^ from xylem) and *SOS1* (role in Na^+^ efflux) and downregulation of *PtHKT2*;*1* (involved in Na^+^ absorption in roots) to ameliorate salt stress in host plants (Niu et al., 2016). Improved physiological performance led to salt tolerance in *B. amyloliquefaciens* SQR9-treated maize plants and correlated to significant increases in the expression of *RBCS* and *RBCL* (RuBisCo subunits), ion transporters (*HKT1*, *NHX1*, and *NHX2*), and *H(C)-Ppase* (encoding HC pumping pyrophosphatase). However, the expression of *NCED* (encoding 9-cisepoxycarotenoid dioxygenase) was downregulated in RI seedlings (Chen et al., 2016). Increased expression of ionic transporters, such as *TaNHX* and *TaHKT1*, and the salt-induced stress gene *TaST* (reduces intracellular Na^+^ level, raises K^+^ content) was observed in PGPR-primed plants relative to NI plants. In addition, upregulated expression of the ABA-responsive gene (*TaABARE*), 12-oxophytodienoate reductase, or *TaOPR1* (enhances antioxidant response) induced expression of *TaMYB* and *TaWRKY*, which led to expression of stress-related genes, including *TaST*. Plant tolerance to salt stress correlated with higher gene expression levels of *CAT*, *APX*, *MnSOD*, *POD*, *GPX*, and *GR*, which together modulated the antioxidant defense system to ultimately confer salt tolerance on inoculated host plants (Bharti et al., 2016). Volatile organic compounds generated by *B. subtilis* reduced gene expression of the high-affinity K^+^ transporter (*HKT1*) in the roots of inoculated *Arabidopsis* plants to limit Na^+^ uptake by the roots (Zhang et al., 2008). Higher transcriptional levels of genes involved in ABA signaling (*RD29A* and *RD29B*), ROS quenching (*APX2*), and detoxification (*GLYI7*) were reported in *Burkholderia phytofirmans* PsJN-colonized *Arabidopsis* plants. Additionally, expression of (*LOX2*) or *Lipoxygenase2* (involved in JA biosynthesis) was downregulated; however, expression patterns of ion transport genes were varied between roots and shoots. *Arabidopsis* K^+^ Transporter 1 or *AKT1* (plasma membrane transporter responsible for K^+^ uptake in roots) expression levels decreased in roots and rosettes after 24 h of salt stress. Sodium Hydrogen Exchanger 2 or *NHX2* (a vacuolar antiporter engaged in ion compartmentalization) was upregulated in roots at all time points, but its expression varied at different points depending on salt stress and bacterial inoculation status. Salt Overly Sensitive 1 or *SOS1* is another plasma membrane Na^+^/H^+^ antiporter (involved in Na^+^ removal from the cytoplasm). Bacterial inoculation caused upregulation of *SOS1* expression in roots after 2 h, and expression was then downregulated after 24 and 72 h. However, in rosettes, increased expression levels were detected after 2 and 24 h. Bacterial inoculation downregulated the expression of High-Affinity *K*^+^ Transporter 1 or *HKT 1* (sodium transporter) in roots under salt stress. Moreover, expression of *HKT 1* was upregulated in the rosettes of non-stressed and colonized plants at 24 h but was downregulated at 24 and 72 h regardless of bacterial inoculation status (Pinedo et al., 2015). *Arthrobacter woluwensis* AK1-treated soybean plants exhibited upregulated expression of various genes, including *GmLAX1* (auxin resistant 1), *GmAKT2* (potassium channel), *GmST1* (salt tolerance 1), and *GmSALT3* (salt tolerance-related gene on chromosome 3) whereas downregulated expression of the ion transporter genes *GmNHX1* (chloride channel gene) and *GmCLC1* (Na^+^/H^+^ antiporter) was observed (Khan et al., 2019). A significant increase in the expression of stress related genes, such as *CAPIP2* (aquaporin), stress related *CaKR1*, *CaOSM1* (osmotin), and *CAChi2* (Class II chitinase), was observed in PGPR inoculated capsicum plants, resulting in the modulation of various biochemical and physiological mechanisms to alleviate salt stress in plants (Yasin et al., 2018). In MI wheat plantlets, transcriptional studies found upregulated expression of P450s genes (*CYP98A1*, *CYP734A5*, *CYP72A15*, and *CYP710A1*) involved in redox reactions and stress responses, *APX*, and Nicotianamine synthase (*NAS*), which is responsible for iron absorption. Moreover, higher expression of oligopeptide transporters (membrane proteins able to transport different substrates), ATP binding cassette (ABC) transporters (proteins mediating energy-driven transport of various substrates), and *HKT* and *NHX* antiporters conferred salt stress resistance on inoculated plants (Safdarian et al., 2019). Increased expression of *PIP* genes was reported in AMF plants compared to non-AMF plants, which improved root water permeability and ameliorated the effects of salt stress conditions (Aroca et al., 2007; Jahromi et al., 2008). Mycorrhizal colonization boosted the expression levels of three chloroplast genes (*RppsbA*, *RppsbD*, and *RprbcL*) (encoding the larger subunit of rubisco) in leaves and genes involved in ion homeostasis (*RpSOS1*, *RpHKT1*, and *RpSKOR*). Higher expression of *RpSOS1* and *RpHKT1* decreased Na^+^ accumulation, while increased *RpSKOR* expression improved K^+^ accumulation in the leaves of mycorrhizal plants, leading to higher K^+^/Na^+^ ratios. Additionally, upregulated expression of *RpPIP1*;*1* and *RpPIP1*;*3* was observed in both leaves and roots; however, *RpPIP2*;*1*, and *RpTIP1*;*1* were found to be more highly expressed in the roots of mycorrhizal plants under salt stress (Chen et al., 2017). Higher nitrogen uptake in salt-stressed mycorrhizal colonized wheat plants correlated with increased expression of *NRT1.1* (involved in nitrate uptake in roots); however, the expression of ammonium transporters (*AMT1.1* and *AMT1.2*) was unaffected. In addition, expression levels of genes related to drought stress (*AQP1*, *AQP4*, *PIP1*, *DREB5*, and *DHN15.3*) were lower compared to non-mycorrhizal wheat plants (Fileccia et al., 2017). Mycorrhizal colonization alleviated the reduced expression levels of *RBCL* (involved in photosynthesis) induced by salt stress; however, salinity raised expression levels of *PPH* (responsible for chlorophyll degradation), which was significantly increased after AMF colonization. Furthermore, genes involved in antioxidant defense responses (*APX*, *Cu-Zn SOD*, *CAT*, and *GR*) showed increased expression levels that were further enhanced by mycorrhizal treatment (Ye et al., 2019).

### Proteomics Studies

Microbial colonization under salt stress leads to up- and downregulated expression of various proteins. Proteomic analysis can reveal the protein profile of inoculated plants to decode the STEM. Detected proteins can be utilized for genetic transformation to boost salt tolerance in crops. Molecular studies performed by Alikhani et al. (2013) revealed that proteome trends in *P. indica*-inoculated barley plants were different from those of NI plants under salt stress. The abundance of the protein peroxiredoxins-2E-2 (component of antioxidant defense system) was increased in both PGPR-colonized and NI plants in the presence of 300 mM NaCl; however, the increase in protein level was greater in *P. indica*-primed plants. Higher expression levels of *RBCS* (small chain of RUBISCO) were reported in *P. indica-*colonized plants compared to NI counterparts. Increased expression levels of xyloglucan endotransglycosylase or *XET* (involved in cell wall biosynthesis) and tubulin-folding cofactor A (involved in cell wall division) were observed in inoculated plants; however, there was no change in expression levels in inoculated plants (with 300 mM NaCl). Furthermore, the expression of papain-like cysteine proteases (cell signaling pathways) was reduced in NI plants, whereas its expression levels were increased in inoculated plants, enhancing salt stress tolerance. Proteomic analysis in AI *E. angustifolia* seedlings showed interesting results. Increased expression of a core protein of PS II (D1 precursor processing PSB27) involved in stabilizing the PSII reaction center was found in chloroplasts, and mitochondria showed increased levels of various energy-related proteins, such as NADH dehydrogenase, iron-sulfur protein NADH dehydrogenase, cytochrome C oxidase, and ATP synthase, to provide energy for cellular activities. Phosphoribosyl transferase or *APT* (enzyme in tryptophan synthesis) was also upregulated in response to AMF colonization. Upregulation of four peptidyl prolyl cis-trans isomerases (peptidyl-prolyl cis-trans isomerase (*FKBP12*), peptidyl-prolyl cis-trans isomerase (*CYP18-1*), *FKBP*-type peptidyl-prolyl cis-trans isomerase 5 isoform 1, and peptidyl-prolyl cis-trans isomerase (*FKBP62*), along with four molecular chaperones, prefoldin subunit 1, prefoldin subunit 2, heat shock 70 kDa, and partial and small hsp 17.3 kDa, was observed in mycorrhizal seedlings to enable correct protein folding under salt stress. In this study, upregulated expression of proteins involved in signal transduction, such as G proteins, plasma membrane Ca^2+^ transporter ATPase (*PMCA*), calcium-dependent protein kinases (*CDPKs*), and calmodulin (*CaM*), enhanced Ca^2+^ signaling. Thus, AMF colonization enhanced expression of proteins involved in secondary metabolism, antioxidant defense, and signal transduction to lead to increased salt tolerance in *E. augustifolia* seedlings (Jia et al., 2019).

## Conclusion and Recommendations

Microbially inoculated plants induce STEM to counteract salt stress and enhance plant productivity. STEM can promote nutrient uptake, enhanced WUE and photosynthesis, preservation of ionic homeostasis and osmoprotection, and efficient antioxidant metabolism. In recent years, various studies have reported that plant–microbe interactions can develop STEM in host plants; however, some aspects of this phenomenon remain poorly understood. Future studies should investigate the role of phytohormonal crosstalk (e.g., BR, JA, and strigolactones) in MI plants to understand their role in eliciting stress signals during salt stress. Metabolomic studies should aim to understand the STEM underlying the secondary metabolism in salt-stressed MI plants. There is also a need for studies investigating the nutritional uptake of sulfur in MI plants under salt stress, given its involvement with glutathione and cysteine (involved in ABA synthesis). Moreover, as the cell wall is the first line of defense during salt stress, subsequent studies should target biochemical and molecular alterations relating to the cell wall of MI plants. Insights into the plant immune system triggered in response to microbial partners while under stress conditions should also be addressed to harness plant microbial interactions for agricultural benefits.

## Author Contributions

MK reviewed, wrote, and revised the manuscript.

## Conflict of Interest

The author declares that the research was conducted in the absence of any commercial or financial relationships that could be construed as a potential conflict of interest.
